# Prediction of overall survival in patients with locally advanced pancreatic cancer using longitudinal diffusion-weighted MRI

**DOI:** 10.3389/fonc.2024.1401464

**Published:** 2024-07-18

**Authors:** Anne L. H. Bisgaard, Carsten Brink, Tine Schytte, Rana Bahij, Mathilde Weisz Ejlsmark, Uffe Bernchou, Anders S. Bertelsen, Per Pfeiffer, Faisal Mahmood

**Affiliations:** ^1^ Laboratory of Radiation Physics, Department of Oncology, Odense University Hospital, Odense, Denmark; ^2^ Department of Clinical Research, University of Southern Denmark, Odense, Denmark; ^3^ Department of Oncology, Odense University Hospital, Odense, Denmark

**Keywords:** diffusion-weighted MRI, biomarker, pancreatic cancer, apparent diffusion coefficient, overall survival

## Abstract

**Background and purpose:**

Biomarkers for prediction of outcome in patients with pancreatic cancer are wanted in order to personalize the treatment. This study investigated the value of longitudinal diffusion-weighted magnetic resonance imaging (DWI) for prediction of overall survival (OS) in patients with locally advanced pancreatic cancer (LAPC) treated with stereotactic body radiotherapy (SBRT).

**Materials and methods:**

The study included 45 patients with LAPC who received 5 fractions of 10 Gy on a 1.5T MRI-Linac. DWI was acquired prior to irradiation at each fraction. The analysis included baseline values and time-trends of the apparent diffusion coefficient (ADC) and DWI parameters obtained using a decomposition method. A multivariable Cox proportional hazards model for OS was made using best-subset selection, using cross-validation based on Bootstrap.

**Results:**

The median OS from the first day of SBRT was 15.5 months (95% CI: 13.2-20.6), and the median potential follow-up time was 19.8 months. The best-performing multivariable model for OS included two decomposition-based DWI parameters: one baseline and one time-trend parameter. The C-Harrell index describing the model’s discriminating power was 0.754. High baseline ADC values were associated with reduced OS, whereas no association between the ADC time-trend and OS was observed.

**Conclusion:**

Decomposition-based DWI parameters indicated value in the prediction of OS in LAPC. A DWI time-trend parameter was included in the best-performing model, indicating a potential benefit of acquiring longitudinal DWI during the SBRT course. These findings support both baseline and longitudinal DWI as candidate prognostic biomarkers, which may become tools for personalization of the treatment of patients with LAPC.

## 1 Introduction

Pancreatic cancer is the fourth most common cause of cancer-related death in Europe, with a 5-year survival rate of less than 10% and an increasing incidence (1, 2). Twenty percent of the patients are eligible for curative-intent surgery, which increases the survival rate to about 20% (3, 4). Radiotherapy (RT) can be used to downstage tumours, making them eligible for surgery or as a definitive treatment. The development of image-guided RT (IGRT), especially the recent introduction of hybrid MRI linear accelerators (MRI-Linacs), have made hypofractionated stereotactic body RT (SBRT) well-tolerated (5–7). Still, the best RT treatment for patients with pancreatic cancer remains to be settled. A possible problem in interpreting the result from pancreatic cancer studies is the considerable variation within the studied cohorts, possibly related to different responses to RT. Thus, biomarkers may allow a more informed treatment choice (8). The impact of such biomarkers could be either dose escalation or de-escalation to obtain an optimal balance between survival and toxicity.

One promising candidate for such a biomarker is the apparent diffusion coefficient (ADC) derived from diffusion-weighted MRI (DWI). ADC quantifies the motion of water molecules, which indirectly reflects the tissue microstructure (9). Several groups have investigated the value of ADC in detecting pancreatic cancer (10–14) and the relation between ADC and pathological response (15–17). The correlation between overall survival (OS) and ADC has also been investigated (4, 18, 19), with findings indicating that pre-treatment ADC might contain more predictive information than standard clinical parameters such as age and tumour size. However, the predictive power of these studies was limited. A potential improvement can be sought through the MRI-Linac, where DWI can be feasibly integrated into the workflow in each treatment fraction. This setup allows investigation of whether changes in DWI parameters during the treatment course could add independent information to the prediction of the outcome for the patients.

The current study utilized longitudinal DWI data to investigate how baseline DWI parameters and changes in DWI parameters during the treatment course impact survival prediction in patients with locally advanced pancreatic cancer (LAPC). DWI parameters were derived using a data-driven method recently proposed by Rahbek et al. (20, 21). The potential advantage of data-driven approaches is that there are no initial model assumptions which could lead to biased parameters. The standard ADC based on a mono-exponential model was included for comparison.

## 2 Materials and methods

A multivariable Cox survival model was made to predict OS in patients with LAPC. The model was based on both clinical parameters and parameters derived from DWI. The DWI parameters were extracted from the GTV volume using both a model-based method (ADC) and a model-free decomposition method. Image analysis was performed by persons without knowledge of the clinical data.

A statistical analysis plan (SAP) was created prior to data analysis of the outcome (**Supplementary Materials 3**). The SAP states time to local progression as a second endpoint. However, due to very few local progression events (8), stable statistical models could not be derived, and therefore, time to local progression is not included in the analysis.

### 2.1 Patients and endpoints

The study included patients diagnosed with LAPC, treated with SBRT for downstaging or definitive (consolidation) SBRT. The patients had a primary tumour or local recurrence in the pancreas. No tumours were resectable at the time of diagnosis based on evaluations at multi-disciplinary team conferences (three patients underwent surgery after SBRT). No lymph nodes were involved at the time of SBRT for any of the patients. All patients received induction chemotherapy using various chemotherapy regimens for at least two months prior to SBRT and were treated with five fractions of 10 Gy on a 1.5 T MRI-Linac (Unity by Elekta, Stockholm, Sweden). Only patients without visual artefacts in the GTV regions for at least two treatment fractions were included. The follow-up after SBRT, including clinical examination and CT scan, was scheduled for every three months for two years. Only patients that attended at least the first three-month follow-up were included in the study, due to the initial intention of using local progression as a second endpoint. OS was defined as the time from the first SBRT fraction to death for any cause. Time between diagnosis and the first SBRT fraction was included as a clinical parameter to take into account differences due to different chemotherapy regimens. All patients were prospectively included in the MOMENTUM study (clinicaltrials.gov NCT04075305) (22), although this specific investigation was planned after patient inclusion. The research ethics committee at the local institutional board (26/68031) approved inclusion of data in MOMENTUM, and the Region of Southern Denmark (20/35211) approved storage of data. Informed consent was obtained from all patients.

### 2.2 MRI protocols

Before treatment, T2-weighted MRI (T2W) scans were acquired on a 1.5 T MRI simulator (Ingenia, Phillips). Imaging before each treatment fraction at the MRI-Linac included T2W and DWI scans. The majority of the patients (n=39) were DWI scanned using the b-values 0, 30, 80, 150 and 500 s/mm^2^ (sequence 1), while six patients were scanned using b-values of 0, 20, 60, 100, 300, 800 and 1000 s/mm^2^ (sequence 2) (acquisition details in **Supplementary Tables S1**, **S2**, **Supplementary Materials 1**).

DWI scans were visually inspected for artefacts. In some patients, an alternation of DWI signal between neighbouring slices was observed, likely due to cross-talk between slices. This was reduced by a pre-processing step in which all slices were convolved with the neighbouring slices with a weight of 0.25 and a weight of 0.5 on the current slice (see **Supplementary Figure S6** in **Supplementary Materials 1**).

### 2.3 Region of interest

GTV delineations from the treatment pre-plan (based on T2W from the MRI simulator) were used to measure the volume of the GTV at baseline. The GTV delineations from the daily adapted treatment plans (based on T2W from the MRI-Linac) were the basis for extracting DWI parameters. The GTVs were transferred to DWIs without translational adjustment. A 5 mm margin was added to the GTVs to account for delineation uncertainty and possible misalignment of the GTV between T2Ws and DWIs which may occur due to motion or geometric distortions present in DWI (23). In the following, all DWI information obtained from the “GTV” refers to the GTV with the 5 mm margin.

### 2.4 ADC calculation

ADC maps were calculated using the standard mono-exponential Stejskal-Tanner model (24), using the b-values 150 and 500 s/mm^2^ (DWI sequence 1) and 300 and 800 s/mm^2^ (DWI sequence 2), to adhere as closely as possible to recommendations from the Elekta MRI-Linac Consortium (25). For each patient, a time-trend was extracted using a linear fit of the median ADC within the GTV as a function of fractions. The slope of the fit as well as the ADC value at fraction one were included in the statistical analysis.

### 2.5 Data-driven DWI decomposition

A decomposition method suited for DWI data, the monotonous slope non-negative matrix factorization method (msNMF) recently introduced by Rahbek et al. (20), was used as a data-driven alternative to the model-based ADC analysis. In brief, the DWI signals can be described as a combination of msNMF components, which represent “typical” behaviours of the DWI signals (linear combination with only positive weights). Thus, each DWI voxel (across all b-values) is represented by weights, one for each component.

**Figure 1:**
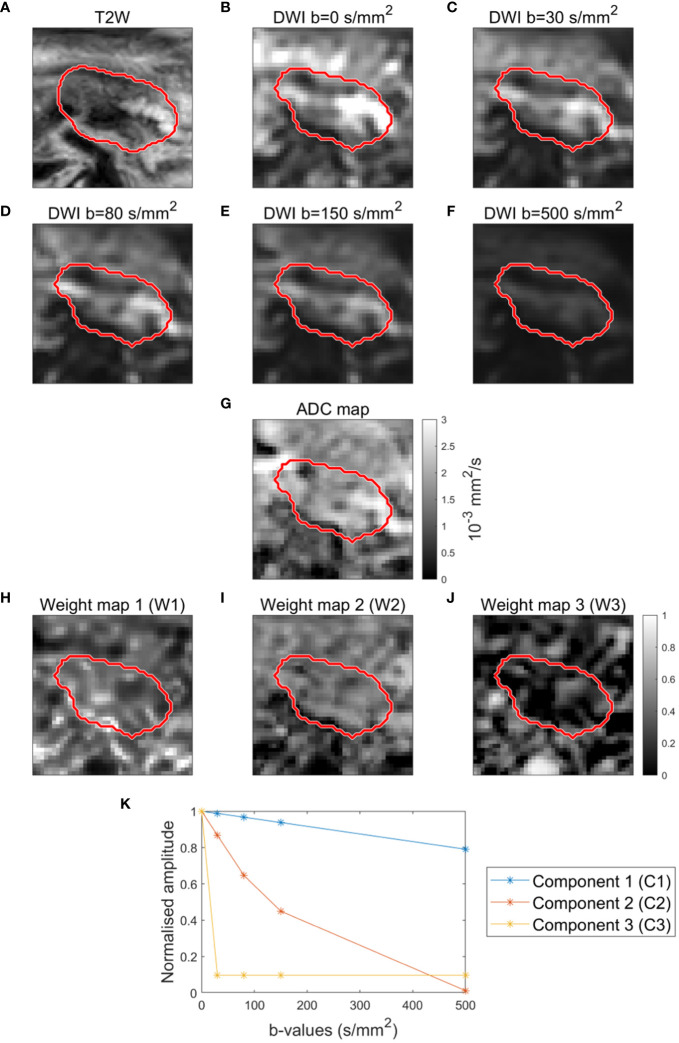
Example of T2-weighted image **(A)**, DWI images **(B–F)** and ADC map **(G)** for a patient. Example of weight maps (W1, W2, W3) corresponding to the three components (C1, C2, C3) **(H–J)**. Components (C1, C2, C3) resulting from decomposition analysis (msNMF) based on a total of 190 DWI scans from 39 patients (5 scans per patient, except for 2 patients with only 2 and 3 scans, respectively) **(K)**.

The components were determined based on data from all patients scanned with DWI sequence 1 (n=39). For this purpose, all GTV voxels from all patients and all fractions were pooled into one data matrix, i.e. only one set of components was derived in total. This set of components was subsequently projected onto each single voxel from all patients (n=45), to obtain the unique weights for each voxel. In the current study, two, three and four components described 86%, 94%, and 96% of the initial data variance, respectively. Based on these values, it was decided to use three components (*k*=3). Thus, in the following, W1, W2 and W3 refer to the weights associated with the components C1, C2 and C3, respectively. The extracted components are shown in **Figure 1** along with example scans. Details regarding image pre-processing and implementation of msNMF in the current study are provided in **Supplementary Materials 2**.

**Figure 2:**
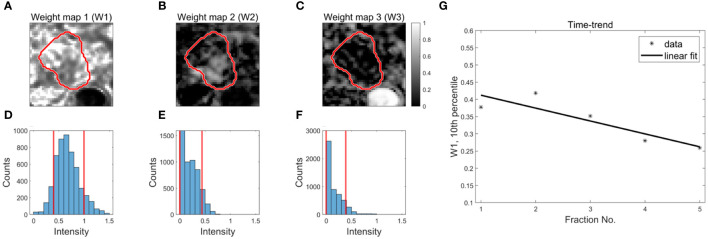
Derivation of decomposition-based DWI parameters for outcome prediction. Examples of weight maps for a patient **(A–C)**. The red contour represents the GTV. The distribution of weights within the GTV (3D volume) are presented using histograms **(D–F)**. The red, vertical lines represent the 10^th^ and 90^th^ percentiles of the distributions. Time trends are extracted from each percentile using a linear fit to the data as a function of fraction number **(G)**.

The spatial distribution of weights associated with the three components were presented as weight maps, (**Figures 2A–C**). To characterize changes in the weights across fractions, the 10^th^ and 90^th^ percentiles of the weight distributions within the GTV were calculated (**Figures 2D–F**). Inspired by Rahbek et al. (21), a time-trend of each percentile was extracted using a linear fit (**Figure 2G**). The slope of the linear fit and the value at fraction one were used as parameters in the statistical analysis. This resulted in 12 decomposition-based parameters [3 components x 2 percentiles x (time-trend + value at fraction one)].

### 2.6 Statistical analysis

A multivariable Cox proportional hazards survival model was made with OS as the endpoint. Patients who were alive at the cut-off date for data collection (March 30, 2023) were censored. In total, 14 DWI parameters (12 decomposition-based and 2 ADC) and 6 clinical parameters were included in the analysis. The clinical parameters at the time of SBRT were age, GTV volume at baseline, time between diagnosis and SBRT, sex, performance status (PS), and primary tumour/recurrence. The continuous parameters were standardized by subtracting the mean and dividing by the standard deviation.

Parameter selection was based on best-subset selection using bootstrap-based cross-validation. This was chosen as a robust method to limit the risk of overfitting compared to e.g. stepwise selection (26, 27). Thus, for each combination of potential parameters for the multivariable model, a bootstrap of the initial data was made. Patients included in the boot (in-boot patients) were used to calculate the regression constants, and the cross-validated model likelihood was subsequently calculated using the patients not included in the boot (out-of-boot patients). Since the number of out-of-boot patients will vary per boot, the computed likelihood was divided by the number of out-of-boot patients, as suggested by Schemper (28). The entire bootstrap process was iterated 50 times, to obtain the mean cross-validated likelihood. The selected multivariable model was the model which performed best during cross-validation. For the best model, model regression constants (*β*) and corresponding 2-sided 95% confidence intervals (CI) defined as the range of the 95% most central values were established using 2000 bootstraps.

The multivariable Cox model was validated using a calibration plot, which allows a visual inspection of how well the model fits the data, as well as the model’s ability to discriminate between patients with at short and long survival time (29). Patients were divided into low-, medium- and high-risk groups and a comparison between the Kaplan-Meier survival curve and model estimate was performed within each risk group. The risk groups were defined based on the model’s ranking of the patients’ survival times (i.e. according to the value of the linear predictor for each patient $\left(\sum_{i}\beta_{i}x_{i}\right)$ , such that the high- and low-risk groups contained the 25% shortest and longest living patients, respectively, and the medium-risk group contained the remaining 50%. Besides the calibration plot, the model’s ability to discriminate between patients with high and low risk was reported using the C-Harrell index, which measures the fraction of patient pairs in which the one with the lower risk survives the longest.

The median survival time for the entire cohort was assessed using the Kaplan-Meier estimator, and the median potential follow-up time was calculated using the reverse Kaplan-Meier method (30). Univariable Cox models were made to provide an overview of the entire data set.

## 3 Results

In total, 50 patients fulfilled the inclusion criteria (see section “Patients and endpoints”). Out of these, five patients were excluded before statistical analysis, as their DWI sequences differed substantially from those of the remaining patients (sequence 1 and 2). For two patients, DWIs were missing for two and three fractions, respectively, however, they were kept as part of the analysis. The analysis was thus based on 45 patients (demographics shown in **Table 1**).

**Table 1:** Patient demographics for 45 patients receiving SBRT for localized pancreatic cancer.

	Overall (N=45)
Age (years)	
Mean (SD)	67.5 (10.1)
Median [Min, Max]	69.5 [45.2, 85.3]
Sex	
F	26 (57.8%)
M	19 (42.2%)
Performance status	
0	17 (37.8%)
1+	28 (62.2%)
Clinical presentation	
Primary tumour (LAPC)	37 (82.2%)
Local recurrence	8 (17.8%)
Time between diagnosis and RT (months)	
Mean (SD)	7.40 (5.89)
Median [Min, Max]	6.48 [1.15, 35.4]
GTV volume (cm^3^)	
Mean (SD)	21.6 (15.8)
Median [Min, Max]	19.1 [2.68, 77.0]
T-stage	
T3-4	45 (100%)
Chemotherapy	
Yes	45 (100%)

The reported T-stage is at the time of diagnosis. No re-staging was performed at the time of recurrence. The reported GTV volumes were obtained from the treatment pre-plan, without the 5 mm expansion.

The decomposition analysis (msNMF) resulted in three well-separated components: one slowly decaying component (C1), one fast decaying component (C2), and one component mainly driven by b=0 s/mm^2^ (C3) (**Figure 1K**). The weight maps revealed tissue heterogeneity, highlighting different regions within the GTV (**Figures 1H–J**). The weight maps did not correlate 1:1 with the DWI images or the ADC map and thus provide complementary information (**Figures 1B–G**).

**Figure 3:**
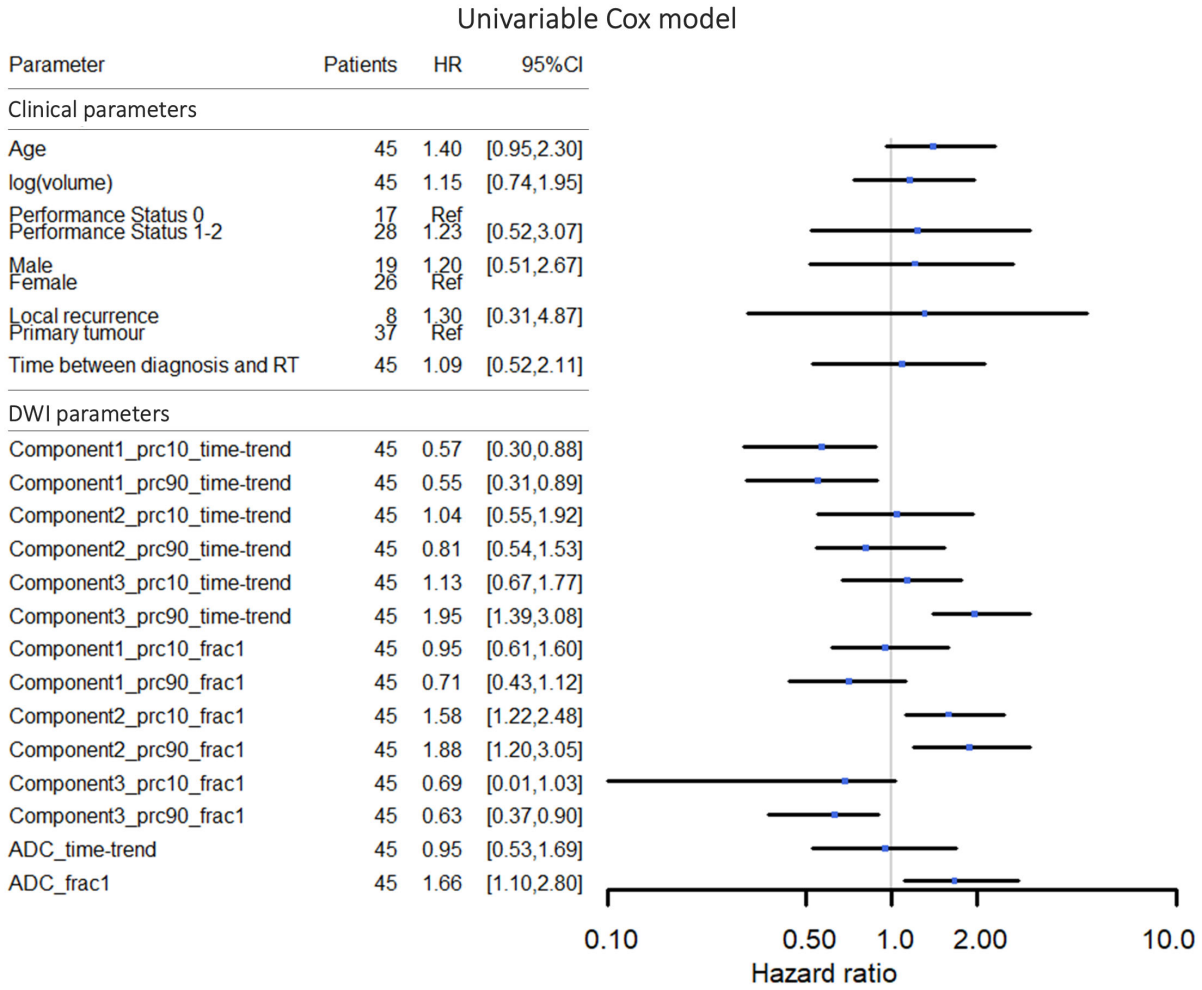
Hazard ratios and 95% confidence intervals for univariable Cox proportional hazards models for overall survival. In the graphical representation, the confidence interval for the DWI parameter “Component3_prc10_frac1” was cut off at 0.10 in order to make a more well-balanced figure. The DWI parameter names refer to the msNMF components, the percentile (10^th^ or 90^th^ percentile), and whether the value represents the time-trend or the value at fraction one (see section “Data-driven DWI decomposition”).

The median OS was 15.5 months (95% CI: 13.2-20.6) (see Kaplan-Meier plot in **Supplementary Figure S1** in **Supplementary Materials 1**). The median potential follow-up time was 19.8 months. Based on univariable analyses, none of the clinical parameters showed a statistically significant association with OS on a 5% level; however, the association between age and OS was borderline significant (**Figure 3**). Several of the DWI-derived parameters, including ADC at the first treatment fraction, showed a statistically significant association with OS.

**Figure 4:**
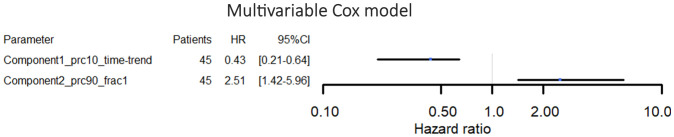
Hazard ratios and 95% confidence intervals for the multivariable Cox proportional hazards model for overall survival. The parameters included in this model were selected by the cross-validation process.

**Figure 5:**
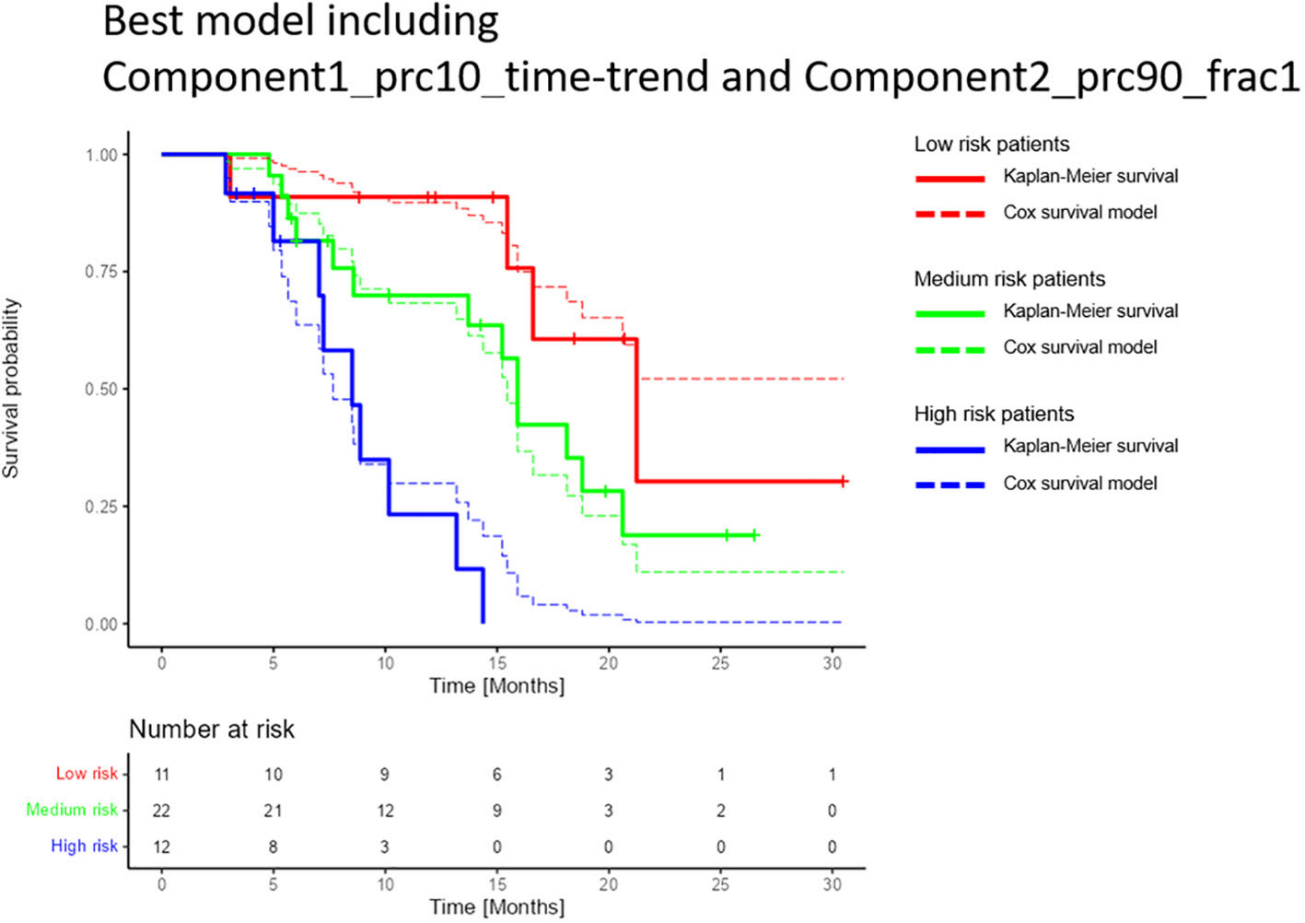
Comparison of the multivariable Cox model and the Kaplan-Meier estimator for the best-performing model for overall survival. The model included the decomposition-based parameters “Component1_prc10_time-trend” and Component2_prc90_frac1” (see section “Data-driven DWI decomposition”). Patients were split into high, medium, and low-risk groups based on the 25% and 75% percentiles of the calculated linear predictors (-0.66 and 0.72), i.e. the high- and low-risk groups each contained 25% of the patients, and the medium-risk group contained 50% of the patients. The confidence intervals for each of the risk groups were overlapping (not shown). Only patients who attended the first three months follow up were included in the study, hence the graphs show a constant survival probability of one during the first three months.

The best-performing cross-validated multivariable Cox model included two decomposition-based parameters (“Component1_prc10_time-trend” and “Component2_prc90_frac1”) (**Figure 4**). The C-Harrell conformance index for the best model was 0.754. A calibration plot demonstrated good agreement between the model and data for all risk groups (**Figure 5**). The individual performance of the two predictors in the best-performing model is shown in **Supplementary Figure S2** in **Supplementary Materials 1**. The model’s performance is notably reduced when the parameters are used separately and have C-Harrell indexes of 0.633 and 0.688 for Component2_prc90_frac1 and Component1_prc10_time-trend, respectively.

## 4 Discussion

This study investigated the value of longitudinal DWI in the prediction of OS in patients with LAPC utilizing parameters derived using both a standard, model based approach (ADC) and a model-free decomposition approach (msNMF). To our knowledge, this study is the first to utilize longitudinal DWI for this purpose.

The best-performing model for OS prediction included only two parameters; one time-trend DWI parameter (Component1_prc10_time-trend) and one baseline DWI parameter (Component2_prc90_frac1), both of which were based on DWI decomposition. None of the clinical parameters were selected by the cross-validation process. The best model reached a C-Harrell index of 0.754 indicating that the model is good at determining which of two patients will survive the longest (a value of 0.5 corresponds to a random guess, and a value of 1 means that the model can perfectly rank the patients’ survival times). The C-Harrell index was markedly reduced if the model was based on just one of the parameters compared to the best model (**Supplementary Figure S2** in **Supplementary Materials 1**), indicating that both baseline information (Component2_prc90_frac1) and longitudinal changes (Component1_prc10_time-trend) might be important for the prediction of OS.

Previous studies have indicated that image-based parameters derived from pre-treatment CT, PET and MR imaging can provide prognostic information in pancreatic cancer (4, 31, 32). With the ability to perform daily imaging in a non-invasive and radiation-free manner, MRI-Linacs represent an interesting opportunity for exploring changes in image-based parameters over the course of treatment using longitudinal imaging. The feasibility of extracting such changes with an MRI-Linac have been demonstrated, with results indicating an increased performance compared to parameters derived at a single time-point (33–35). The current study made use of this opportunity to investigate image-based parameters derived from longitudinal DWI, exploring different methods for extraction of parameters. The msNMF method has previously been tested in patients with brain metastasis, showing borderline significant differences between responders and non-responders (21), but has not been tested as a predictor of OS in pancreatic cancer. It was included in the current study to investigate whether it could provide information useful for the prediction of OS in pancreatic cancer, as a proof of concept. Interestingly, the cross-validation process selected decomposition-based parameters instead of ADC information. A possible explanation could be that the decomposition-based parameters might be more stable than the standard ADC values. Increased stability may be achieved as the extracted components are based on data from all patients and fractions, which may reduce the impact of noise. The C-Harrell index for a model based on the baseline ADC (ADC_frac1) and the ADC time-trend (ADC_time-trend) was 0.623 (data not shown), indicating an inferior discriminating power. That being said, the Pearson correlation between ADC_frac1 and Component2_prc90_frac1 is 0.78, showing that most of the information in Component2_prc90_frac1 is also present in the ADC signal (**Supplementary Figure S4** in **Supplementary Materials 1**). Thus, based on the current results, the main difference between the standard ADC approach and the msNMF approach is within the longitudinal changes in which msMNF parameters showed a stronger association with OS compared to ADC_time-trend. The potential added benefit of the msNMF approach compared to the ADC approach should be weighed against the increased complexity of the image post-processing, which may make msNMF more labour intensive.

The multivariable Cox model shown in **Figure 4** suggests that high values of Component2_prc90_frac1 are associated with reduced expected lifetime (hazard ratio (HR)>1). According to the univariable Cox models presented in **Figure 3**, the same applies for high values of ADC_frac1 (HR>1), which is in line with the observed correlation between Component2_prc90_frac1 and ADC_frac1 (**Supplementary Figure S4** in **Supplementary Materials 1**). Initially, these results seem to contradict the findings in some other studies, in which an association between low baseline ADC (comparable to ADC_frac1 in the current study) and poor survival was reported (18, 19, 36). The explanation might be a difference in the definition of the region of interest from which the DWI parameters were derived. The current study used the clinically available GTV so that no additional delineation was needed, whereas previous studies focused on the delineation of the “viable” part of the tumour, which differs from the GTV by excluding necrotic and cystic parts. This explanation is supported by a study by Lyng et al. (37), which shows that some necrotic regions are related to increased ADC values. Furthermore, necrosis has been related to less favourable outcomes in patients with LAPC (38), which matches high values of ADC_frac1 in patients with low OS seen in the current study. That ADC’s capacity as a biomarker of response is affected by the choice of regions of interest has also been demonstrated in other anatomical regions (39).

For Component 2 frac1 and Component 1 time-trend, the 10% and 90% percentiles contain quite similar information, with a correlation of 0.70 and 0.61, respectively (**Supplementary Figure S4** in **Supplementary Materials 1**). Since the average of the 10% and 90% percentile values for symmetrical distributions is very close to the median, it is likely that the median values could have been used instead. In fact, a model based on the related median values of the best model (i.e. a model based on “Component1_prc50_time-trend” and Component2_prc50_frac1”) had a C-Harrell index of 0.757 (**Supplementary Figure S5** in **Supplementary Materials 1**), similar to the C-Harrell index of the best model (0.754). For future studies, it is likely as good (and simpler) to focus on the median values instead of the tails of the weight distributions.

To take into account the heterogeneity of the patient cohort, clinical parameters such as primary tumour/local recurrence were included in the analysis. Interestingly, the cross-validation process did not select any of the clinical parameters, indicating a superiority of baseline and longitudinal DWI to predict OS compared to standard clinical parameters.

It should be noted that the two parameters in the best model showed a low correlation with the clinical parameters. The largest Pearson correlation between the model parameters and the continuous clinical parameters was 0.24, and the lowest p-value of a Mann–Whitney U test of best model parameters between the levels of the categorical clinical parameters was 0.53. Therefore, there is no indication that the DWI parameters could be a proxy for any of the clinical parameters.

In general, OS may depend on many factors, both related to the tumour and to the individual patient’s response to treatments, including SBRT. The observed association between DWI parameters and OS could potentially be related to differences in the tumour microstructure between patients, which might be captured by DWI. While ADC is commonly interpreted in terms of cell density, decomposition-based parameters could potentially reflect the composition of the tumour microstructure, as sub-compartments within each image voxel may contain different tissue types. A possible explanation for the observed association between DWI parameters and OS could be that the tumour microstructure is indicative of the tumour aggressiveness and tendency to form distant metastasis, something that is likely linked to OS (18). Moreover, tumours with different microstructure may respond differently to treatments. Based on the results, it might be relevant to investigate the predictive value of DWI, in order to identify patients who responds well/poorly to SBRT.

Initially, time to local progression was included as a second endpoint in the statistical analysis plan. However, due to a small number of local progression events (n=8), it was not possible to derive stable statistical models, and thus, no results are presented for this endpoint. Hence, it might be a relevant topic for a future study to investigate if DWI could provide information of the local response to a local treatment (SBRT), as this information might be relevant in order to personalize the treatment.

A careful parameter selection based on cross-validation was performed to limit the effect of overfitting, instead of e.g stepwise parameter selection (26, 27). However, it is still considered a major limitation of the current study that the statistical model was based on 45 patients only. Although it is considered a relatively large cohort compared to other RT studies within LAPC, it may be a poor representation of patients with LAPC treated worldwide. Validation of the statistical model in patients from other centres is thus warranted.

In conclusion, a statistical model to predict survival after SBRT in patients with LAPC based on only two DWI parameters has been developed. The model contains both baseline information and DWI changes during the SBRT course and showed ability to discriminate between patients with short and long survival times. It is the hope that in the future, prognostic information from DWI can assist in stratifying patients for individual treatment (e.g. dose escalation), to improve treatment outcomes.

## Data availability statement

The data analysed in this study is subject to the following licenses/restrictions: The data are not publicly available. Informed written consent was obtained from all patients to include the data in the Momentum study (a database where data is shared between hospitals in the Elekta MRI-linac Consortium). Requests to access these datasets should be directed to The Momentum data management task force (https://mrlconsortium.org/introduction-to-momentum/).

## Ethics statement

The studies involving humans were approved by Research Ethics Committee at the University of Southern Denmark. The studies were conducted in accordance with the local legislation and institutional requirements. Written informed consent for participation in this study was provided by the participants’ legal guardians/next of kin.

## Author contributions

ABi: Conceptualization, Data curation, Formal analysis, Investigation, Project administration, Software, Visualization, Writing – original draft, Writing – review & editing. CB: Conceptualization, Formal analysis, Investigation, Resources, Software, Writing – original draft, Writing – review & editing. TS: Conceptualization, Investigation, Resources, Supervision, Writing – review & editing. RB: Investigation, Resources, Writing – review & editing. MWE: Conceptualization, Investigation, Resources, Writing – review & editing. UB: Investigation, Writing – review & editing. ABe: Investigation, Writing – review & editing. PP: Resources, Writing – review & editing. FM: Conceptualization, Funding acquisition, Project administration, Supervision, Writing – review & editing.
