# Supplementary material for: Prediction of overall survival in patients with locally advanced pancreatic cancer using longitudinal diffusion-weighted MRI
Source: Front Oncol. 2024 Jul 18;14:1401464. doi: 10.3389/fonc.2024.1401464 (PMC11291378; doi:10.3389/fonc.2024.1401464)
Supplement: Supplementary file 1 [file DataSheet_1.docx]

# Supplementary materials 1: Figures and Tables

## Table S1 and Table S2: MRI acquisition parameters

Table S1: Acquisition parameters for T2-weighted images acquired on both MRI-Linac and the MRI simulator.

| **T2W:** | MRI-Linac | MRI simulator |
| --- | --- | --- |
| Sequence | 3D SE | 3D SE |
| Fat saturation | No | No |
| TE/TR (ms) | 137/1400 | 137/1400 |
| Parallel imaging (SENSE factor) | 3.7 | 3.7 |
| No. of excitations (NEX) | 3 | 3 |
| In-plane resolution (mm) | 1x1 | 1x1 |
| Field of view (mm) | 448x448 | 488x488 |
| Slice thickness (mm) | 2 | 2 |
| Slice gap (mm) | 0 | 0 |
| Scan duration | 6 min and 4 s | 6 min and 4 s |

Table S2: Acquisition parameters for DWI-sequences on the MRI-Linac

| **DWI:** | Sequence 1 (39 patients) | Sequence 2 (6 patients) |
| --- | --- | --- |
| Sequence | Monopolar diffusion encoding, 2D SE with single shot EPI readout | Monopolar diffusion encoding, 2D SE with single shot EPI readout |
| Diffusion gradient encoding | Three orthogonal directions along the imaging plane axes | Three orthogonal directions along the imaging plane axes |
| Fat saturation | SPIR | SPIR |
| Number of signals averaged (NSA) | 1 | 4 |
| b-values (No. of excitations (NEX)) | 0 (2)  30 (2)  80 (2)  150 (4)  500 (12) | 0 (1)  20 (1)  60 (1)  100 (1)  300 (1)  800 (3)  1000 (3) |
| Gradient duration (ms) ($\delta$) | 61.72 | 71.78 |
| Effective diffusion time (ms) $\left( \Delta-\frac{\delta}{3} \right)$ | 60.64 | 60.83 |
| TE/TR (ms) | 82.30/3354.17 | 84.72/628.58 |
| Parallel imaging (SENSE factor) | 2 | 2 |
| In-plane resolution (mm) | 1.92x1.92 | 1.22x1.22 |
| Field of view (mm) | 224x224 | 288x288 |
| Slice thickness (mm) | 4 | 6 |
| Slice gap (mm) | 0.00 | 0.60 |
| Scan duration | 4 min and 11 s | 3 min and 56 s |

## Figure S1: Kaplan-Meier plot for overall survival

**
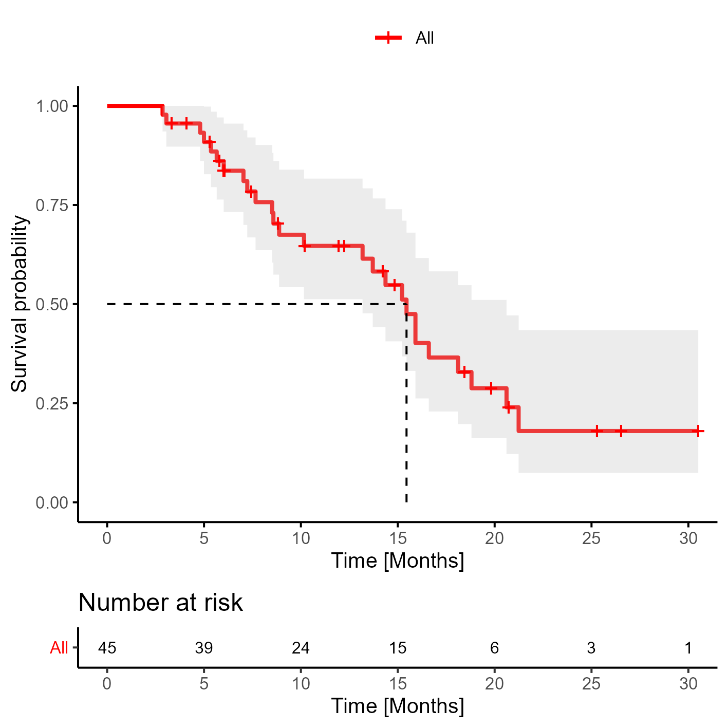
**

Figure S1: Kaplan-Meier plot for overall survival. The red curve represents the Kaplan-Meier estimator, while the shaded area represents the 95% confidence interval. The dotted line represents the median survival time.

## Figure S2: Univariable models for the parameters included in the best-performing model


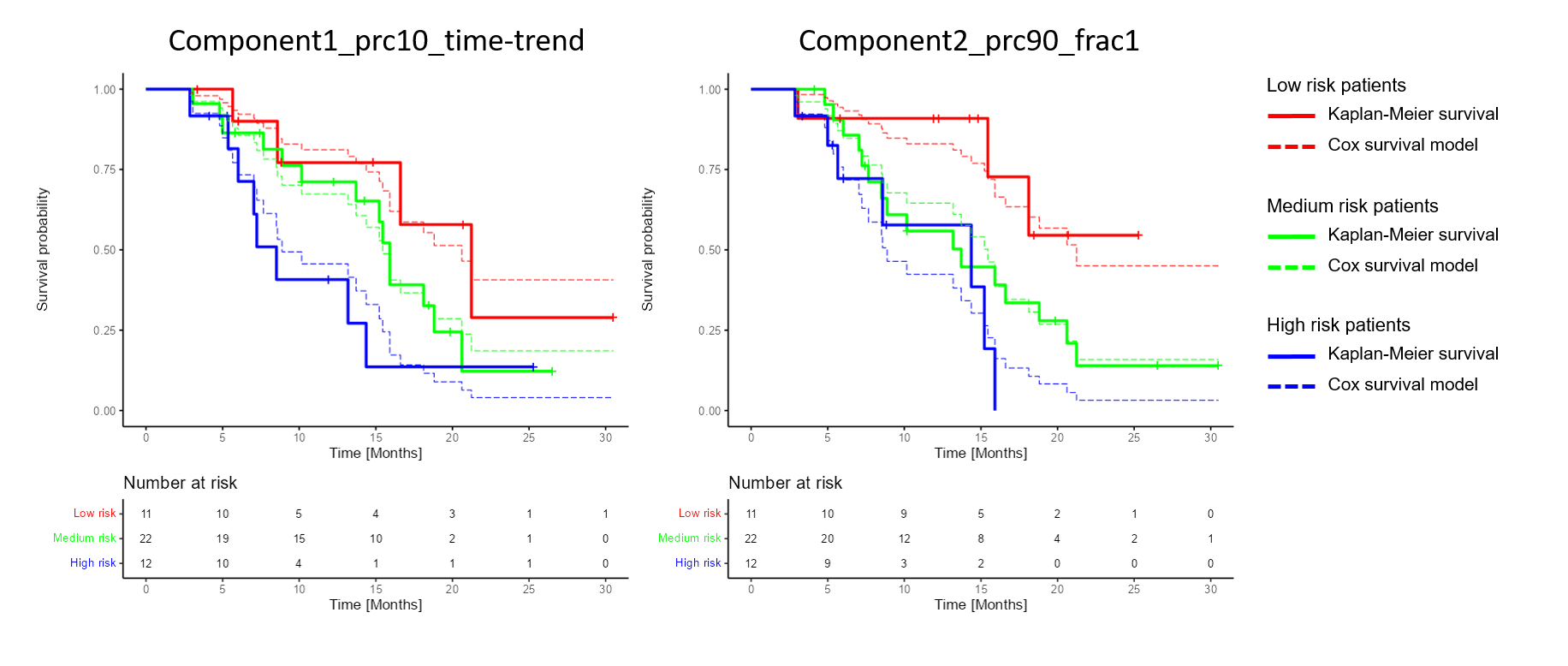


Figure S2: Comparison of the univariable Cox model and the Kaplan-Meier estimator for the parameters "Component1_prc10_time-trend" and "Component2_prc90_frac1" respectively. Patients were split into high, medium and low-risk groups based on the 25% and 75% percentiles of the calculated linear predictors, i.e. the high and low-risk groups each contained 25% of the patients, and the medium-risk group contained 50% of the patients. The division-coefficients of the linear predictor were -0.32 and 0.42 for "Component1_prc10_time-trend" and -0.43 and 0.47 for "Component2_prc90_frac1".

## Figure S3: Univariable model for ADC


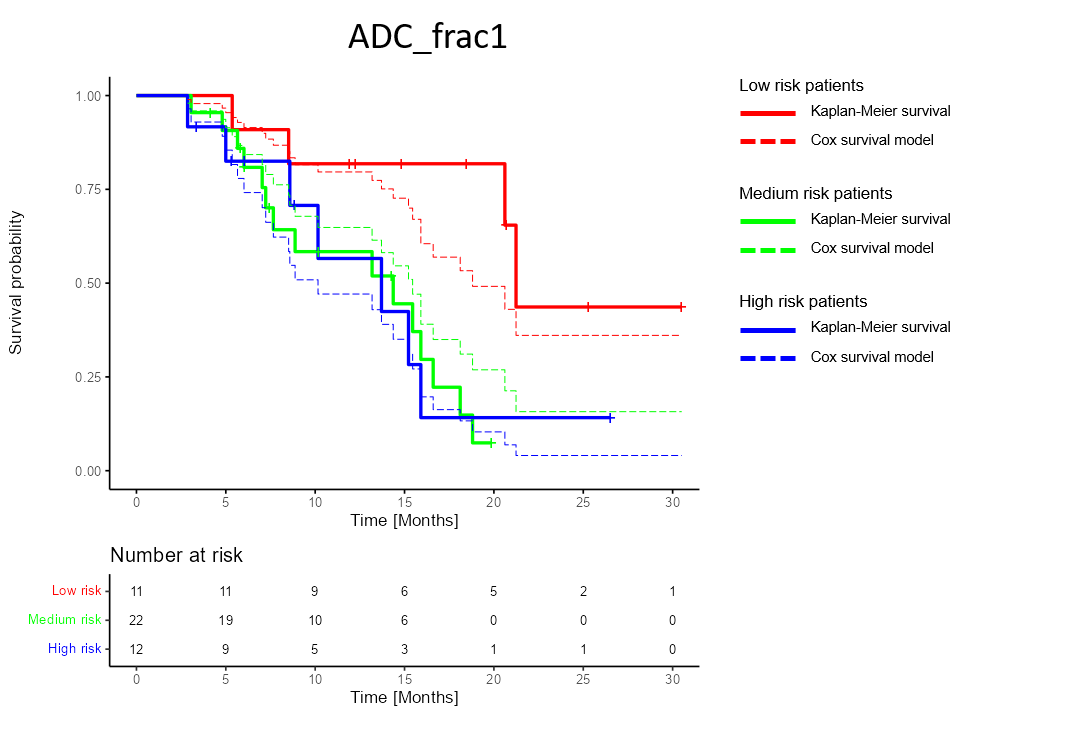


Figure S3: Comparison of the univariable Cox model and the Kaplan Meier estimator for ADC at fraction 1. Patients were split into high, medium and low-risk groups based on the 25% and 75% percentiles of the calculated linear predictors, i.e. the high and low risk groups each contained 25% of the patients, and the medium-risk group contained 50% of the patients. The division-coefficients were -0.52 and 0.37.

## Figure S4: Correlation between DWI parameters


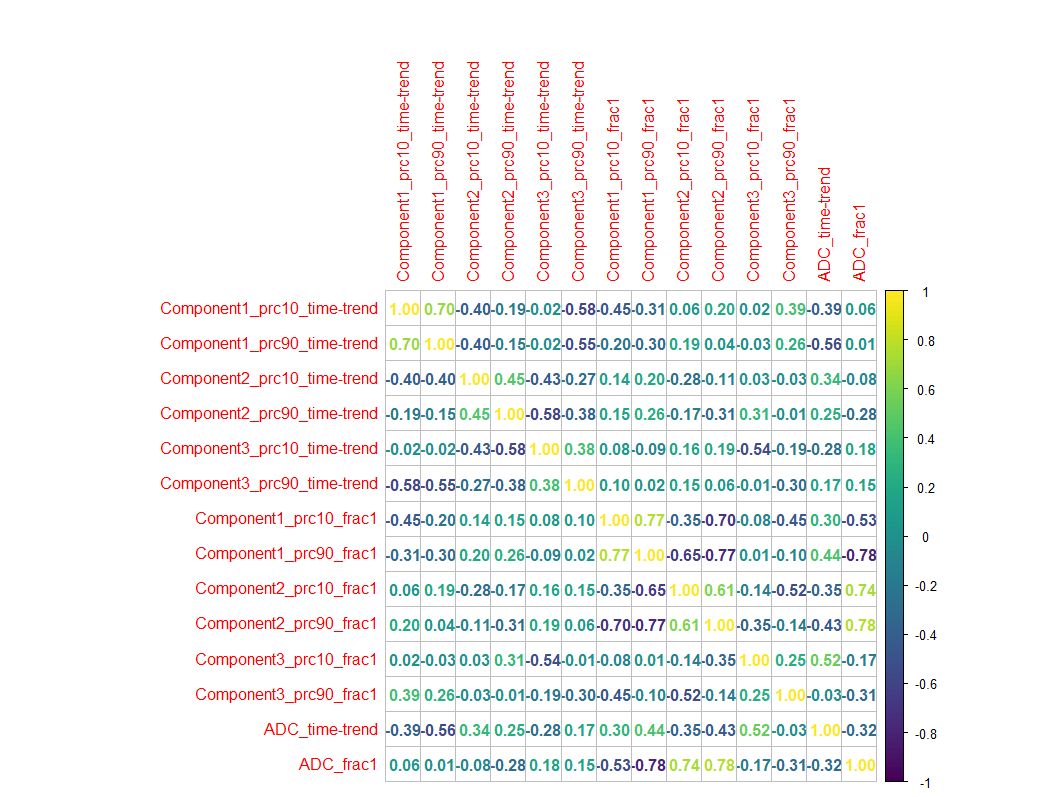


Figure S4: Pearson's correlation coefficient for all pairs of DWI parameters.

## Figure S5: Multivariable model including median values


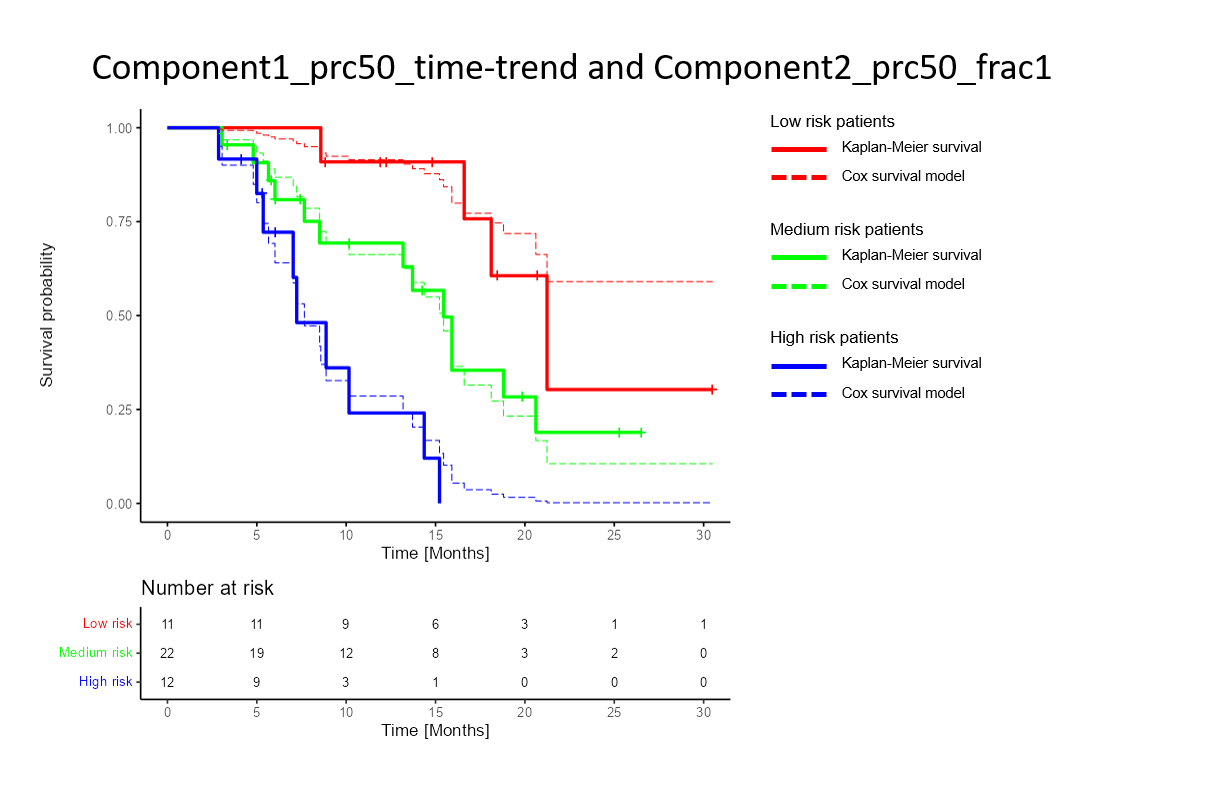


Figure S5: Comparison of the multivariable Cox model and the Kaplan Meier estimator for "Component1_prc50_time-trend" and "Component2_prc50_frac1". Patients were split into high, medium and low-risk groups based on the 25% and 75% percentiles of the calculated linear predictors, i.e. the high and low-risk groups each contained 25% of the patients, and the medium-risk group contained 50% of the patients. The division-coefficients were -0.54 and 0.80.

## Figure S6: Correction of DWI images


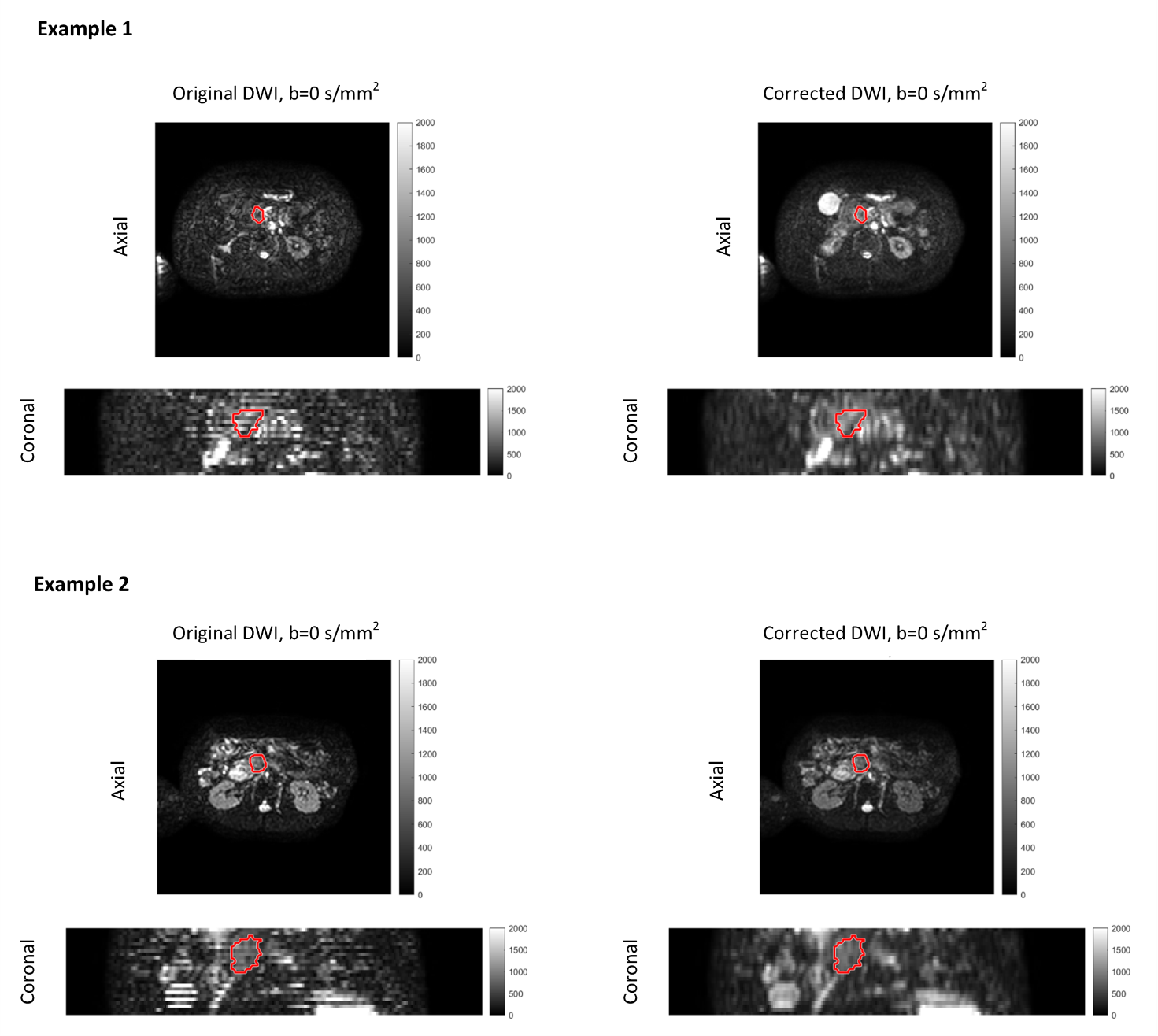


Figure S6: Example of an original DWI scan showing alternating DWI signal between slices (left), and the same image after it has been corrected using a convolution of the signal between neighboring slices (right). The red contour represents the GTV.

# Supplementary materials 2: Image pre-processing and implementation of msNMF

**Image pre-processing**

The DWI signal within each voxel was normalized by dividing by the signal intensity at b=0 s/mm^2^, as the signal behavior is of interest and not the absolute signal intensity. Overall, DWI signals should decrease as a function of the b-value, however, image noise can disturb the measured signal between b-values, particularly for voxels with low signal values. To reduce the impact of noise, voxels with normalized signal values above 1 for b$\geq$80 s/mm^2^ were excluded before calculating the msNMF components. Further, voxels were excluded if a linear fit to the logarithm of the signal had a positive slope (voxels with a non-decreasing signal). As a result, 22% of the voxels were excluded based on this noise reduction approach. Distributed across all DWI scans, the excluded voxels constituted in median 15% (range 0-71%) of the GTV voxels.

**Implementation of msNMF**

To determine $\boldsymbol{C}$ and $\boldsymbol{W}$**,** the Frobenius norm of the residual, $\left\| \boldsymbol{X}-\boldsymbol{CW} \right\|_{F}^{2}$, was minimized under the constraints that $\boldsymbol{W}$ and $\boldsymbol{C}$ are non-negative, and that the components and the slopes of the components are monotonous. Minimization was performed using an alternating non-negative least squares (ANLS) algorithm, where $\boldsymbol{C}$ was optimized while $\boldsymbol{W}$ was kept constant and vice versa.

A stochastic gradient descent framework was used to solve the non-convex optimization problem. $\boldsymbol{X}$ was divided into bathes of 1000 voxels each, and for each batch, $\boldsymbol{C}$ was initialized as the $\boldsymbol{C}$ resulting from the previous batch while a random initialization was used for $\boldsymbol{W}$. The optimization continued until convergence. Data was shuffled between each epoch, i.e. each batch did not contain the same voxels for each epoch. Based on the resulting components, $\boldsymbol{C}$, a final optimization of $\boldsymbol{W}$ was performed for each patient while keeping $\boldsymbol{C}$ constant, i.e. by solving $\min_{\boldsymbol{H}\geq0} \left\| \boldsymbol{X-CW} \right\|_{F}^{2}$. This was done both for patients scanned with DWI sequence 1 and 2. For patients with DWI sequence 2, $\boldsymbol{C}$ was interpolated/extrapolated to match the b-values in DWI sequence 2 ranging from 0-800 s/mm^2^. The b-value of 1000 s/mm^2^ in DWI sequence 2 was excluded from the analysis, due to the noise floor present for high b-values (b>=1000 s/mm^2^).

# Supplementary materials 3: Statistical analysis plan

## **Statistical analysis plan**

**Title:**Prediction of response in pancreas tumours using parameters derived from longitudinal diffusion-weighted MRI

**SAP number with dates:**
Version 2, date: 27.06.2023

**Protocol version:**

Project description of the Ph.D. project

| “DW-MRI as a decision making tool in the in-room MRI guided radiotherapy pipeline “ updated on the 10.01.2022 by Anne Bisgaard. See “Study 3”. |
| --- |

**SAP revision:** This is the second version of the SAP. This SAP was completed on the 27.06.2023, before any analysis was performed on the outcome variables.

**Roles and contributions:**

Author: Anne Bisgaard, Ph.D.-student. Laboratory of Radiophysics, Department of Oncology, Odense University Hospital.

Main supervisor: Faisal Mahmood, Ph.D. Laboratory of Radiophysics, Department of Oncology, Odense University Hospital.

Co-supervisor: Carsten Brink, Ph.D. Laboratory of Radiophysics, Department of Oncology, Odense University Hospital.

Co-supervisor: Tine Schytte, Ph.D. Department of Oncology, Odense University Hospital.

This plan is based on the TRIPOD Checklist for Prediction Model Development, item 1-3 and item 6a through 11. (<http://www.tripod-statement.org/TRIPOD/TRIPOD-Checklists/TRIPOD-Checklist-Prediction-Model-Development>)

**Title and abstract**

**Item 1. Title**

Prediction of response in pancreas tumours using parameters derived from longitudinal diffusion-weighted MRI

**Item 2. Abstract**

**Introduction**

**Item3.a. Explain the medical context (including whether diagnostic or prognostic) and rationale for developing or validating the multivariable prediction model, including references to existing models.**

Pancreas cancer is the fourth most common cause of cancer-related death in the western world [1]. The 5-year survival rate is less than 10%[2]. The treatment include surgery, chemotherapy and radiotherapy (RT). Some patients have surgery after RT, however not all patients are suited for surgery. In any case, down-staging RT is used to achieve local control.

Prediction of response to neo-adjuvant treatment would allow for treatment adaptation, which could potentially improve the outcome or reduce toxicity for the individual patient. The rationale for developing the prediction model is to allow treatment adaptation based on information from quantitative MRI. The model is prognostic.

DWI is a potential biomarker for response to neo-adjuvant treatment for patients with pancreas cancer [3]. Typically, the “apparent diffusion coefficient” (ADC) is derived from DWI images using a mono-exponential model [4]. However, using models may lead to biased parameters, as they do not take into account partial volume effects. To overcome this problem, data driven approaches may be preferred. An example is the newly developed monotonous slope non-negative matrix factorization (ms-NMF), which uses a decomposition of the DWI signal to extract parameters[5]. The aim of this study is to test whether these parameters can be used to predict outcome in terms of time to local progression based on the RECIST criteria[6] and overall survival in patients with pancreas cancer treated with RT.

**Item 3b. Specify the objectives, including whether the study describes the development or validation of the model or both.**

Hypothesis:

- Parameters derived from longitudinal DWI acquired on the MR-linac can be used to predict time to local progression based on the RECIST criteria and overall survival in patients with pancreas cancer

**Methods**

**Item 6a. Clearly define the outcome that is predicted by the prediction model, including how and when assessed.**

The outcome is time to local progression based on the RECIST criteria and overall survival. According to the RECIST criteria, the patients are categorized in the following way, based on the longest tumour diameter (LD) at baseline and at follow up [6]:

- Complete response (CR): no target volume left at follow up
- Partial response (PR): at least 30% decrease in LD compared to baseline
- Progressive disease (PD): at least 20% increase in LD compared to baseline
- No change: no change in LD compared to baseline
- Stable disease (SD): the patient does not qualify for any of the above statements

The above categories are chosen based on changes in both the tumour and lymph nodes. Hence, a patient may be categorized as a PR even if the tumour diameter does not change between baseline and follow up. Therefore, in addition to the above categories (CR, PR, PD or SD), we also collect information about whether the progression is local or distant, based on the tumour diameter to be able to evaluate the response of the tumour alone using the RECIST criteria.

The following information is collected:

- The time for diagnosis (primary tumour or recurrence)
- The time for the start of the RT treatment.
- The time of progression (PD), either local *or* distant, based on the RECIST criteria (based on both tumour and lymph nodes).
- Local or distant progression? This is determined based on the RECIST criteria (based on tumour alone).
- The time of resection of the tumour.
- The time of death.

The endpoint is:

- Time to local progression, defined as the time between start of RT to the time of local progression.
- Overall survival, defined as the time from start RT to death.

The censoring criteria, when looking at time to local progression is:

- Distant progression (if the patient has distant progression, it is not possible to know if the patient also has local progression).
- Tumour resection
- Death
- Patient moved to another hospital
- None of the above and no progression at the cut-off date (30/03 2023)

The censoring criteria, when looking at overall survival is:

- Patient is alive at the cut-off date (30/03 2023)
- Patient moved to another hospital

**Item 6b. Report any actions to blind assessment of the outcome to be predicted.**

No actions have been taken to blind assessment of the predictors for the outcome and other predictors. This is not expected to influence the data, as none of the predictors or endpoinds depend on subjective decisions.

**Item 7a. Clearly define all predictors used in developing the multivariable prediction model, including how and when they were measured.**

| Predictor | Measured how and when |
| --- | --- |
| Tumour GTV at baseline (cm^3^) | Measured using MRI from Ingenia at baseline. |
| Primary tumour or local relapse | Whether the tumour being treated with RT is a local relapse or primary tumour. |
| DWI derived parameters | Parameters derived using the msNMF method developed by Sofie Rahbek. |

| Sex | M/F |
| --- | --- |
| Age at time of inclusion |  |
| Time from diagnosis to RT start |  |
| Performance status before RT start | Measured 0-4 weeks before RT |

**Note**: All patients had a T-stage>=T3, and all patients received chemotherapy before RT (some patients also received chemotherapy after RT, and this might lead to differences in the outcome, however, it is not possible to obtain these data).These are important factors to predict outcome, however, since they are identical for all patients, they are not included in the model.

**Item 7b. Report any actions to blind assessment of predictors for the outcome and other predictors.**

No actions have been taken to blind assessment of the predictors for the outcome and other predictors. This is not expected to influence the data, as none of the predictors or endpoints depend on subjective decisions.

**Item 8. Explain how the study size was arrived at.**

All patient data meeting the eligibility criteria available from our institution by the beginning of end of March 2023 was evaluated to reach as high statistical power as possible. The eligibility criteria are:

- Primary tumour or relapse in pancreas, adenocarcinoma
- Treated with 5 fractions radiotherapy on MRL
- All five fractions were delivered
- DWI scans were acquired from at least one fraction
- The image quality of DWI scans is acceptable based on visual inspection.
- Follow up data is available for at least the 3 months follow up scan. I.e. patients who started RT before the 1^st^ of January 2023 are included.

**Item 9. Describe how missing data were handled (for example, complete-case analysis, single imputation, multiple imputation), with details of any imputation method.**

| **Predictor** | **n missing** | **Reason for missingness – missing completely at random (MCAR)?** | **Handling of missing values** |
| --- | --- | --- | --- |
| Tumour GTV volume at baseline (cm^3^) | 0 |  |  |
| DWI derived parameters | 5 scans | Missing for some fractions if DWI was not acquired. | If at least two fractions are available for a patient, the slope and intercept from a linear fit will be used. |
| Primary tumour or local relapse | *0* |  |  |
| Sex | 0 |  |  |
| Age | 0 |  |  |
| Time from diagnosis to RT start | 0 |  |  |
| Performance status before RT start | 0 |  |  |

**Item 10a. Describe how predictors were handled in the analyses.**

| **Predictor** | **Scale/unit** | **Coding** | **Test of coding** |
| --- | --- | --- | --- |
| Tumour GTV volume | cm^3^ | Continous |  |
| DWI derived parameters  Chosen based on best subset selection using CV | mm^2^/s | Continous |  |
| Primary tumour or local relapse | Primary tumour or local relapse | Primary tumour: 0  Local relapse: 1 |  |
| Sex | M/F | Factor:  Male: 0  Female: 1 |  |
| Age | Years | Continous. |  |
| Time from diagnosis to RT start | Months | Continous |  |
| Performance status before RT start | 0, 1, 2 | 0, 1, 2 |  |

**Item 10b. Specify type of model, all modelbuilding procedures (including any predictor selection), and methods for internal validation.**

The prediction framework is based on a decomposition of the DWI signal into components. This method produces “mixture maps”, showing the weights of each component for each voxel. The 10th and 90th percentiles of each component within the GTV (plus a 5 mm margin) are extracted. A linear fit is performed for each percentile as a function of fraction number. The slope of this fit as well as the value at fraction 1 are used as predictors. Likewise, the slope and value at fraction one of the median ADC are included.

Apart from the above-mentioned DWI predictors, 6 clinical predictors were included: tumour GTV volume at baseline, relapse (yes/no), sex, age, time from diagnosis to RT and performance status are included. Predictor selection is the best subset method utilizing bootstraping for cross-validation

- All subsets of variables are tested.
- For each subset, a Cox proportional Hazard model is build and tested using bootstrapping.
- The best-performing model in terms of the cross-validated likelihood is chosen
- Seedpoint used for bootstrapping: 42
- Number of bootstraps: 50

**Item 10d. Specify all measures used to assess model performance and, if relevant, to compare multiple models.**

One model will be chosen for each endpoint based on maximal likelihood.

**Item 11. Provide details on how risk groups were created, if done.**

**Referencer**

[1] Kirkegård J, Bojesen AB, Nielsen MF, et al. Trends in pancreatic cancer incidence, characteristics, and outcomes in Denmark 1980–2019: A nationwide cohort study. Cancer Epidemiol. 2022;80:102230.

[2] McGuigan A, Kelly P, Turkington RC, et al. Pancreatic cancer: A review of clinical diagnosis, epidemiology, treatment and outcomes. World J Gastroenterol. 2018;24:4846–4861.

[3] Beaton L, Bandula S, Gaze MN, et al. How rapid advances in imaging are defining the future of precision radiation oncology. Br J Cancer. 2019;120:779–790.

[4] Stejskal EO, Tanner JE. Spin diffusion measurements: Spin echoes in the presence of a time-dependent field gradient. J Chem Phys. 1965;42:288–292.

[5] Rahbek S, Madsen KH, Lundell H, et al. Data-driven separation of MRI signal components for tissue characterization. J Magn Reson. 2021;333:107103.

[6] Villaruz LC, Socinski MA. The clinical viewpoint: Definitions, limitations of RECIST, practical considerations of measurement. Clin Cancer Res. 2013;19:2629–2636.
